# Fabrication and Applications of Potentiometric Membrane Sensors Based on Specific Recognition Sites for the Measurement of the Quinolone Antibacterial Drug Gemifloxacin

**DOI:** 10.3390/molecules28135144

**Published:** 2023-06-30

**Authors:** Gamal A.E. Mostafa, Essam A. Ali, Rashad A. Alsalahi, Haitham Alrabiah

**Affiliations:** Department of Pharmaceutical Chemistry, College of Pharmacy, King Saud University, P.O. Box 2457, Riyadh 11451, Saudi Arabiahalrabiah@ksu.edu.sa (H.A.)

**Keywords:** gemifloxacin, supramolecular chemistry, potentiometry, sensors, PVC sensors, drug formulation and quality control

## Abstract

Supramolecular gemifloxacin (GF) sensors have been developed. Supramolecular chemistry is primarily concerned with noncovalent intermolecular and intramolecular interactions, which are far weaker than covalent connections, but they can be exploited to develop sensors with remarkable affinity for a target analyte. In order to determine the dose form of the quinolone antibacterial drug gemifloxacin, the current study’s goal is to adapt three polyvinylchloride (PVC) membrane sensors into an electrochemical technique. Three new potentiometric membrane sensors with cylindric form and responsive to gemifloxacin (GF) were developed. The sensors’ setup is based on the usage of o-nitrophenyl octyl ether (o-NPOE) as a plasticizer in a PVC matrix, β-cyclodextrin (β-CD) (sensor 1), γ-cyclodextrin (γ-CD) (sensor 2), and 4-tert-butylcalix[8]arene (calixarene) (sensor 3) as an ionophore, potassium tetrakis (4-chlorophenyl) borate (KTpClPB) as an ion additive for determination of GF. The developed method was verified according to IUPAC guidelines. The sensors under examination have good selectivity for GF, according to their selectivity coefficients. The constructed sensors demonstrated a significant response towards to GF over a concentration range of 2.4 × 10^−6^, 2.7 × 10^−6^, and 2.42 × 10^−6^ mol L^−1^ for sensors 1, 2, and 3, respectively. The sensors showed near-Nernstian cationic response for GF at 55 mV, 56 mV, and 60 mV per decade for sensors 1, 2, and 3, respectively. Good recovery and relative standard deviations during the day and between days are displayed by the sensors. They demonstrated good stability, quick response times, long lives, rapid recovery, and precision while also exhibiting good selectivity for GF in various matrices. To determine GF in bulk and dose form, the developed sensors have been successfully deployed. The sensors were also employed as end-point indicators for titrating GF with sodium tetraphenyl borate.

## 1. Introduction

**Figure 1:**
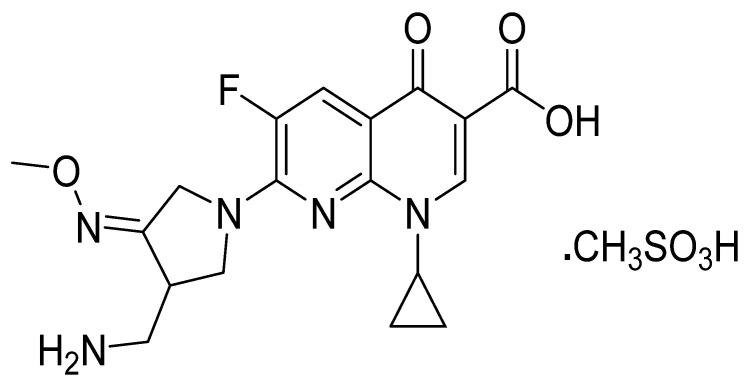
Structure of gemifloxacin mesylate.

Gemifloxacin mesylate (GF) is a broad-spectrum quinolone antibacterial agent for oral administration:(*Z*-7-[3-(aminomethyl)-4-(methoxyimino)-1-pyrrolidinyl]-1-cyclopropyl-6-fluoro-1,4-dihydro-4-oxo-1,8-naphthyridine-3-carboxylic acid monomethine sulfonate (C_18_H_20_FN_5_O_4_·CH_4_O_3_S) (Figure 1). It is a fluoroquinolone antibiotic that destroys bacteria and prevents DNA synthesis by inhibiting both topoisomerase IV and DNA gyrase [1,2]. It is effective against both Gram-negative and Gram-positive bacteria and is used to treat infections caused by susceptible bacterial strains [3,4,5].

According to a literature review, analytical methods used for the determination of gemifloxacin include spectrophotometry [6,7,8], spectrofluorometry [9,10], polarography [11], voltammetry [12,13,14], HP-TLC [15,16], HPLC-UV [17,18,19]^,^ HPLC-F [20,21,22], HPLC-MS [23,24,25], and potentiometry [26,27,28,29].

The majority of reported methods demand pricey equipment and sample pretreatments. Potentiometric techniques based on membrane sensors are simple, affordable, precise, quick to respond, and unaffected by the presence of samples with color or turbidity [29]. The published potentiometric methods [26,27,28] for GF measurement were based on the utilization of coated wire [26], ZnO nanorod [27], and ion-pair as electroactive materials in PVC matrix [28]. Ionophores were used as the sensing materials in the current investigation, which focused on host–guest interaction. The examined sensors’ selectivity is higher for ionophore-based method than it is for ion-pair technique, whereas the membrane sensors selectivity is dependent on the complexation or inclusion reaction between host and guest.

**Scheme 1:**
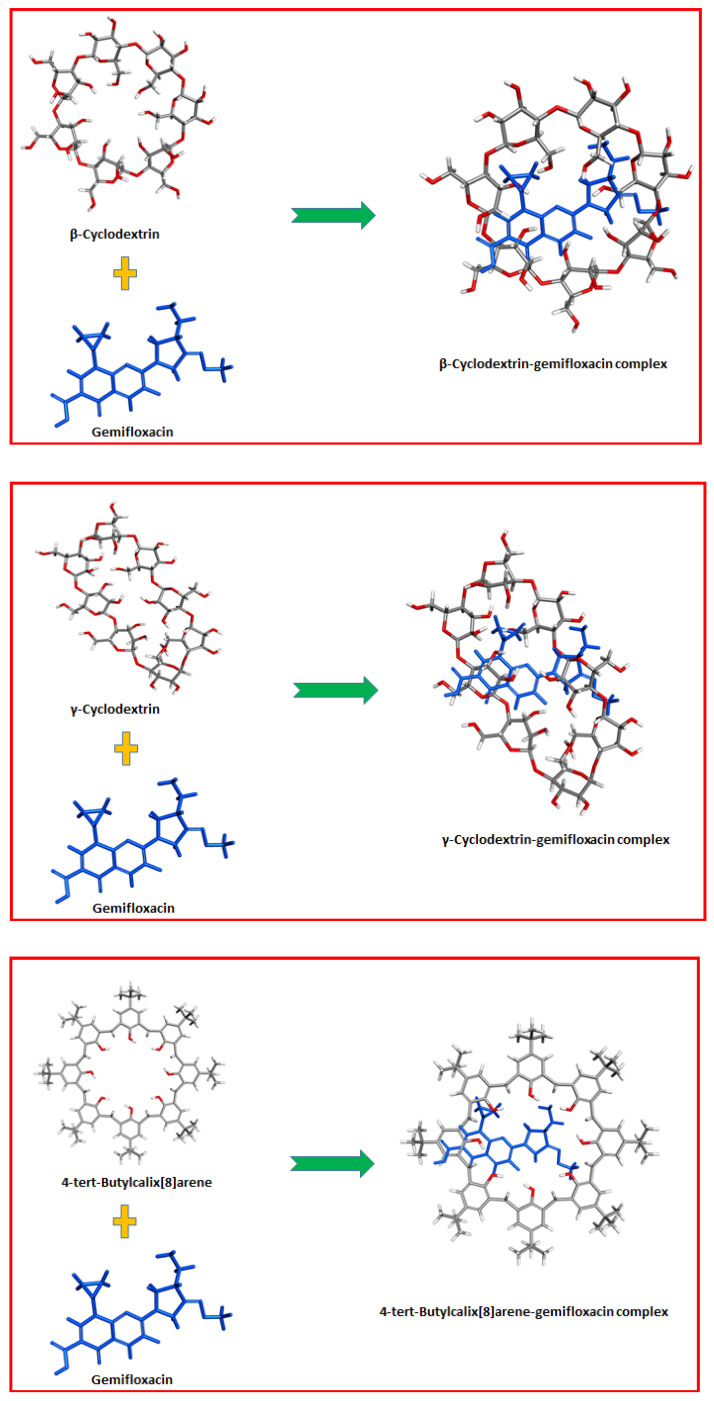
A diagram of GF, and their complex with β-CD, γ-CD, and calixarene.

Numerous studies have focused on the use of potentiometric ion-selective electrodes to assess distinct active ingredient drugs that employ various cyclodextrins (CD) and calixarene modifiers based on the host–guest inclusion process [30,31]. Non-covalent interactions between the guest and host are required for inclusion complex formation. A growing focus in host–guest and supramolecular chemistry is on molecular identification and inclusion complexations as key chemical sensing techniques [32,33]. The majority of the cooperative binding with particular guest molecules was attributed to intermolecular hydrogen bonding between the CD molecules, hydrophobic interactions, and van der Waals forces between the host and guest molecules also played a role in the cooperative binding processes [30] (Scheme 1). Through dipole–dipole interactions, calixarenes are well known for acting as selective ligands for various ions. They can bind to a variety of cation substrates to produce stable host–guest inclusion complexes. Numerous cation-selective electrodes have been produced using the property of calixarenes [31] (Scheme 1). Additionally, the donor atoms oxygen and nitrogen found in GF support the coordination relationship between the host and guest. However, the positive charge of gemifloxacin facilitates the coordination of the guest and host processes by producing an inclusion complex reaction.

The current work is distinctive in that it employs ionophores (β-CD, γ-CD, and calixarene), which appear to offer stronger sensor qualities in terms of selectivity, more linear response, cost-effectiveness as well as accuracy, and long-life. The goal of this study is to develop a unique modulated ion-selective sensor for GF detection in pharmaceutical formulations using calixarene, β-CD, and γ-CD as molecular recognition hosts in the presence of KTpClPB as an ion additive. The developed techniques were subsequently employed as quality control tools to identify gemifloxacin in bulk and dose form. In light of IUPAC standards for analytical processes and requirements, the method was validated.

## 2. Results and Discussion

### 2.1. Characterization of the Membrane Sensors

#### 2.1.1. Effect of the Additive and Ionophore

The complexes formed between the host and the analyte were charged, but this charge was neutralized by the addition of an ion-exchanger or lipophilic ion. The type of ion-exchanger or lipophilic ion was chosen in accordance with the type of analyte [34,35] which neutralized the charge between host and guest. In this investigation, we employed the lipophilic ion KTpClPB, which reduced anionic interferences and improved the selectivity for the drug gemifloxacin while neutralizing the charge produced between the GF and host [34,35]. As a result, we used KTpClPB as a lipophilic ion to improveme selectivity by neutralizing the charge created by the inclusion complex while also acting as an anionic excluder. The sensitivity and selectivity toward the target analyte (GF) are thereby enhanced by the addition of lipophilic ions [34,35]. The ionophore to lipophilic ion (KpCTPB) ratio of was 5:1 (*w*/*w*%). The examined sensors’ selectivity and sensitivity were enhanced by the addition of 5 mg of (KpCTPB). The engagement was predicated on the understanding of host–guest interaction. The developed sensors, which are based on host–guest interactions, exhibit a significant affinity for gemifloxacin with strong selectivity and Nernstian response in the presence of lipophilic ions.

The impact of the ion exchanger and ionophore concentration on the electrode response was investigated. The effectiveness of the suggested sensors was tested using various ion exchanger and ionophore concentrations. Table 1 summarizes the results. The best membrane composition was observed at 5 mg of the ion exchange for sensors 1, 2, and 3, respectively. The electrodes response showed the best characteristics 55 mV/decade, 56 mV, and 60 mV/decade for sensor 1, 2 and 3, respectively. In contrast, the optimal ionophore composition was investigated and it was found that at 25 mg (ionophore), showed the best electrode response at 55 mV/decade, 56 mV/decade, and 60 mV/decade for sensors 1, 2, and 3, respectively. Table 1 shows the results, which demonstrate that the optimal compositions for the ion-exchanger and ionophore were at 5 mg and 25 mg, respectively.

#### 2.1.2. Effect of Plasticizer

Different plasticizers have been studied for the gemifloxacin-PVC membrane sensors (β-CD, γ-CD, and calixarene). Because the dielectric constant of the plasticizer affects the potential response, three electrodes were produced using three different plasticizers, dibutyl phthalate (DBP), dioctyl phthalate (DOP), and o-NPOE. The objective of plasticizers in the formation of PVC membranes is to create a membrane with homogenous properties and flexibility that are mostly dependent on the type of plasticizer used. Both DOP and o-NPOE were shown to be effective plasticizer mediators for gemifloxacin sensors, as shown in Table 1. DOP (ε = 5.1) and o-NPOE (ε = 24) have the necessary solvation properties for the ionophores needed to build the suggested sensors; however, o-NPOE has slightly superior membrane properties than DOP and DBS (ε = 4.5). Where the dielectric constant of o-NPOE is greater than that of DOP, the produced sensors will have marginally better properties than DOP. Table 2 lists the impact of plasticizer type on sensor performance. The response to different plasticizers is shown as follows: Sensor 1 measured 55 mV/decade (DOP), 53 mV/decade (DBP), and 55 mV/decade (o-NPOE), while Sensor 2 measured 55 mV/decade (DOP), 53 mV/decade (DBP), and 56 mV/decade (o-NPOE). Sensor 3 measured 58 mV/decade (DOP), 55 mV/decade (DBP), and 60 mV/decade (o-NPOE). Results show that o-NPOE performed better than DOP and DBP. As a result, all incoming studied o-NPOE data are utilized.

### 2.2. Effect of pH and Response Time

**Figure 2:**
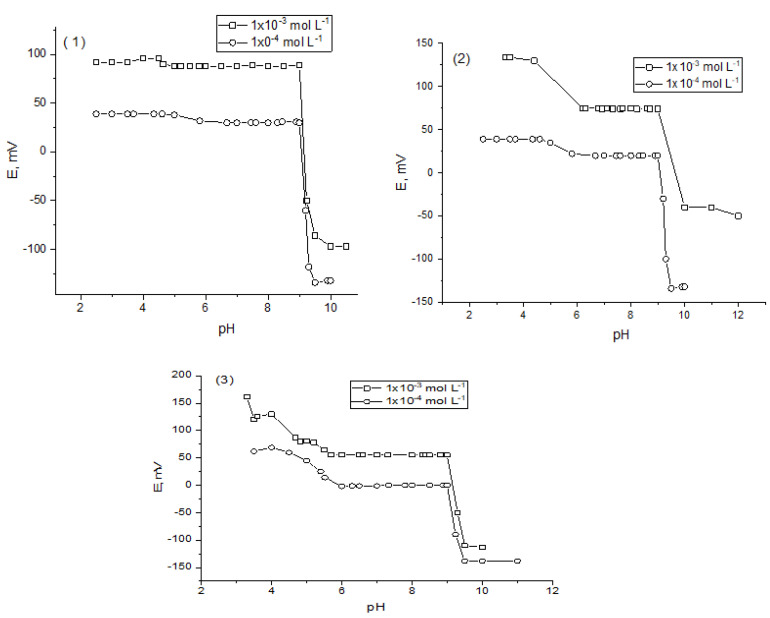
The effect of pH on the potentiometric response of gemifloxacin sensors (**1**): β-CD (**2**) γ-CD, and (**3**) calixarene sensors.

The influence of pH on various GF concentrations was investigated across a wide range of pH ranging from 2–12 in order to produce the optimum pH. The ideal circumstances for the influence of pH on various GF concentrations were investigated throughout a wide pH range ranging from 2 to 12. A highly diluted solution of HCl and NaOH was used to measure the pH. The GF selective sensors were evaluated at two concentrations of the GF, 0.001 M and 0.0001 mol L^−1^, at various pH levels. The potential of the investigated sensors was consistent between pH levels of 6 and 9 (Figure 2). The developed sensors show that the slope interval is constant at the optimal pH range (55, 56, and 60 mV per decade for sensors 1, 2, and 3, respectively). The most accurate measurement solution seemed to be an acetate buffer solution at pH 7. The potential decreases when the un-protonated form of GF grows at increasing pH (pH > 9) (pKa = 9 [36]). The pH range of 6 to 9 had no effect on the potential. The time necessary to obtain a steady state potential (1 mV) after increasing the GF concentration over the calibration graph concentration was utilized to determine the response times of the construct sensors. At 20 s, the developed sensors exhibit a stable potential reading over the calibration concentration range. The PVC membrane sensors have a lifespan of around 8 weeks, during which time their potential has been consistent, and they have shown good accuracy and precision.

### 2.3. Interference Studies

The gemifloxacin sensors were tested with a variety of organic and inorganic particles. The potentiometric selectivity coefficients were calculated in an acetate buffer at pH 7 using an IUPAC recommendation using either separate solution or mixed solution method. The following Equations (1) and (2) [37,38] was used to compute the selectivity coefficient ($K_{A,\;B}^{Pot})$ of the recommended sensors.

(1) $$\log K_{A,\;B}^{pot}=\;\;\frac{E_{B-}E_{A}}{S}+\left[1-\frac{Z_{A}}{Z_{B}}\;\;\right]\log a_{A}$$

where $(a_{A}$ and $a_{B})$ and (*Z*_A_ and *Z*_B_) are the activities and charges of GF and interfering species, respectively, and S is the slope of the graph “mV/decade”. The potential readings for GF and interfering ion concentrations (0.001 M each) are *EA* and *EB.* The mixed solution method was used to calculate the selectivity coefficient using Equation (2):

(2) $$K_{A,\;\;B}^{pot}\;\;=\frac{\left({a^{\prime }}_{A}-a_{A}\right)}{a_{B}}$$

where “${a^{\prime }}_{A}$” is the known activity of “a primary ion that is added to known solution that contains a fixed activity ($a_{A})$ of primary ions, and the corresponding potential change (ΔE) is recorded. In another test, an interfering ion solution ($a_{B}$) is added to a known solution until the same potential change (E) is observed. Table 3 summarizes the results of the selectivity coefficient. Due to the low selectivity coefficient, the results demonstrated that the suggested procedures were free of influence from the interfering ions.

### 2.4. Validation of the Method

The suggested sensor assays have been validated in accordance with the IUPAC criteria [39]. The linearity range, limit of detection, limit of quantification, robustness, accuracy, and precision are all validated.

#### 2.4.1. Linearity and Range

The relationship between concentration and potential is a logarithmic relationship, according to the Nernstian Equation (3).

(3) $$E=E_{{}^{\circ}}+Slog\left[GF\right]$$

**Figure 3:**
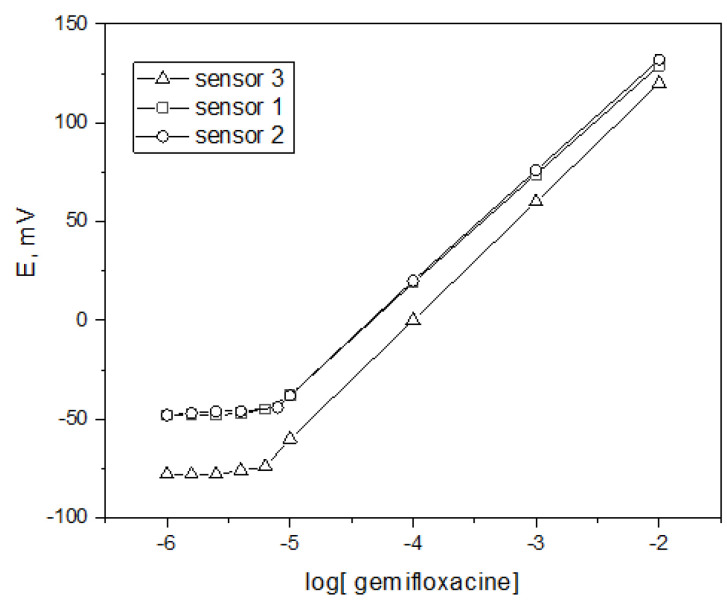
Calibration of gemifloxacin using the proposed sensors (sensor1: β-CD, sensor 2: γ-CD and sensor 3: Calixarene).

E(mV) is the produced potential and ”*E_°_* “ is the standard electrode potential which is presented by the intercept and “*S*” is the slope. Each measurement *E(mV)* was made five times for each sensor, and then the average potential was plotted against log (GF), as presented in Figure 3. The linearity of calibration graph was 1 × 10^−2^ to 8 × 10^−6^ mol L^−1^, 1 × 10^−2^ to 9 × 10^−6^ mol L^−1^ and 1 × 10^−2^ to 8 × 10^−6^ mol L^−1^ for sensors 1, 2, and 3 at the optimum condition. Coefficient of determination, *R*^2^ was 0.999, 0.998, and 0.999, respectively (Table 4)

#### 2.4.2. Limit of Detection/Quantification

According IUPAC recommendation [39], the limits of detection (LOD) were 2.45 × 10^−6^, 2.75 × 10^−6^, and 2.45 × 10^−6^ mol L^−1^ for the investigated sensors 1, 2, and 3, respectively, whereas the limit of quantification (LOQ) was 8×10^−6^ mol L^−1^, 9 × 10^−6^ mol L^−1^ and 8 × 10^−6^ mol L^−1^ for the sensors 1, 2, and 3, respectively.

#### 2.4.3. Accuracy

The accuracy of the suggested sensors has been evaluated, and the results are summarized in Table 5. The accuracy was calculated as percentage recoveries (%R) ± relative standard deviation. The data revealed that more than 97.5% was accomplished using the indicated method either during the day or between days.

#### 2.4.4. Precision

For each proposed sensor assay, intra-day precision (determinations repeated at different times on the same day) and inter-day precision (determinations repeated on three different days) were investigated. Table 4 summarizes the results. The RSD% is less than 2.4% throughout the day and less than 2.7% throughout different days, the data demonstrate good precision.

### 2.5. Comparison of Ionophore with Ion-Pair Based PVC Sensors

Table 6 compares the proposed techniques with the potentiometric technique that has been published. The developed sensors exhibit a near-Nernstian response (55, 56, and 60 mV/decade) in comparison to the non-Nernstian response (20 mV/decade [26] and 33 mV/decade [27]) and are even more sensitive than the previously described method [26]. In contrast, the proposed method was used to measure the GF in aqueous solutions with a controlled pH as opposed to measurements in aqueous solutions with an uncontrolled pH [26,28].

### 2.6. Application of GF Sensors

The results of using the suggested sensors to measure GF in both bulk and dose form are displayed in Table 5. The accuracy with the indicated sensors is 98.5%, 98%, and 99%, but the RSD with sensors 1, 2, and 3 was 2.9%, 2.85%, and 2.80%, respectively. Table 6 shows the results. On the other hand, the suggested method for GF test in its dosage version has been implemented. Table 7 shows the results. The results reveal that there is a high degree of accuracy and precision.

The precision and accuracy of the proposed sensors were statistical compared with published method [40] using the student *T*-test and F-test as presented in Table 8. The results demonstrate that there is no statistically significant difference between the proposed sensors and the reported method with regard to accuracy and precision because the calculated student *T*-test (0.12–0.15) and F-test (1.1–1.2) values were less than their critical values (T tabulated = 3.36 and F tabulated = 6.38) [41]. A one-way ANOVA test with a 95% confidence level was used to statistically evaluate the assay data. The proposed sensors test and the reported method, as shown in Table 7 did not reveal any statistically significant differences, according to the results.

#### Application of GF Sensors as Indictor Electrode

**Figure 4:**
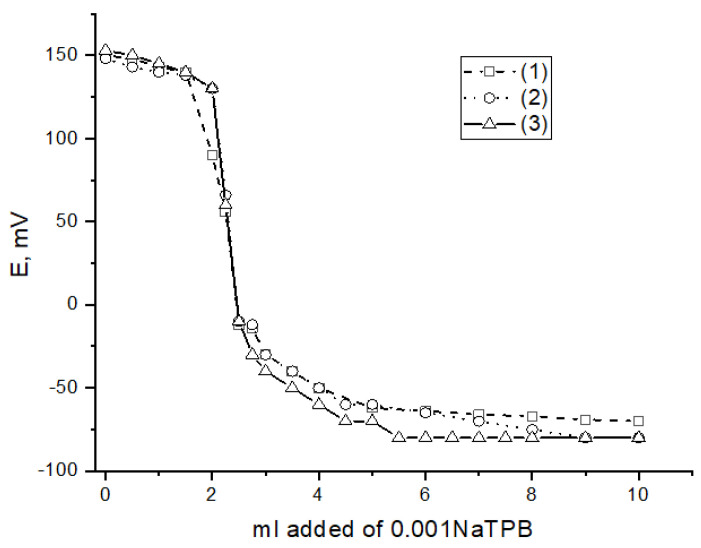
Titration of 2.5 mL of 0.001 M gemifloxacin with 0.001 M NaTPB using sensor (1): β-CD, (2): γ-CD, and (3): calixarene.

The developed sensors have been evaluated as an end point indication electrode in numerous potentiometric titrations with an Ag/AgCl reference electrode. GF solution has been used to titrate sodium tetraphenylborate. GF and sodium tetraphenylborate react in a 1:1 molar ratio, according to the results. The symmetrical titration curves with a very noticeable potential jump of around 150 mV confirmed the high sensitivity of the proposed sensors (Figure 4).

## 3. Experimental

### 3.1. Apparatus

HANNA pH 211 microprocessor pH meter (Made in Europe, Romania) with GF sensors and a calomel reference electrode (Merck) was used for all potentiometric readings, unless otherwise noted, at 25 °C. A combined Ross glass pH electrode (Orion, La Verne, CA, USA) was used to adjust the pH.

### 3.2. Reagents and Materials

All of the compounds were of the analytical grade. All the water was double distilled. Tetrahydrofuran (THF), DBP, DOP, o-NPOE, and high molecular weight PVC powder (code number: 81387 and K-value 69–71) were all purchased from Merck Co. in the Rahway, NJ, USA. The following compounds were obtained from BDH (Poole, UK): GF, β-CD, γ-CD, calixarene, and potassium tetrakis (4-chlorophenyl) borate (KTpClPB). Factive tablets from Tabuk Pharmaceuticals in Riyadh, Saudi Arabia, contain 320 mg of gemifloxacine mesylate. Caffeine, glucose, and starch microcrystalline cellulose were obtained from Merck (Steinheim, Germany). GF was dissolved in water in the proper quantity to create a stock solution (1 × 10^−1^ mol L^−1^). The stock of GF was serially diluted with water to create four workable solutions. Utilizing 0.05 M sodium acetate and acetic acid, an acetate solution with a pH of 7 was made.

### 3.3. Preparation of the GF Sensors

A 5-cm glass Petri dish containing, 5 mg of ion-exchanger, 25 mg ionophore materials (β-CD, γ-CD, or calixarene), plasticizer (DBS, DOP, or o-NPOE) (350 mg), and PVC powder (190 mg) was thoroughly mixed, then THF was then added to the dish [42,43]. After mixing, the solvent was evaporated all night long. According to reported methods [42,43], THF was used to connect the produced PVC membrane to glass electrode bodies. A 0.01 M GF and KCl mixture was used to fill the inner solution of the glass electrode. By soaking the sensors in GF solution and maintaining them there when not in use, the sensors were condition.

### 3.4. Effect of pH and Response Time

The potential (E, mV) was measured in relation to pH variation. To investigate the effect of pH on the characteristic response of the GF sensors, two GF concentrations (1 × 10^−3^ and 1 × 10^−4^ mol L^−^1) were utilized. The pH was changed with a very diluted solution of HCl or NaOH. The potential (E, mV) was measured in relation to pH variation.

### 3.5. Calibration

**Scheme 2:**
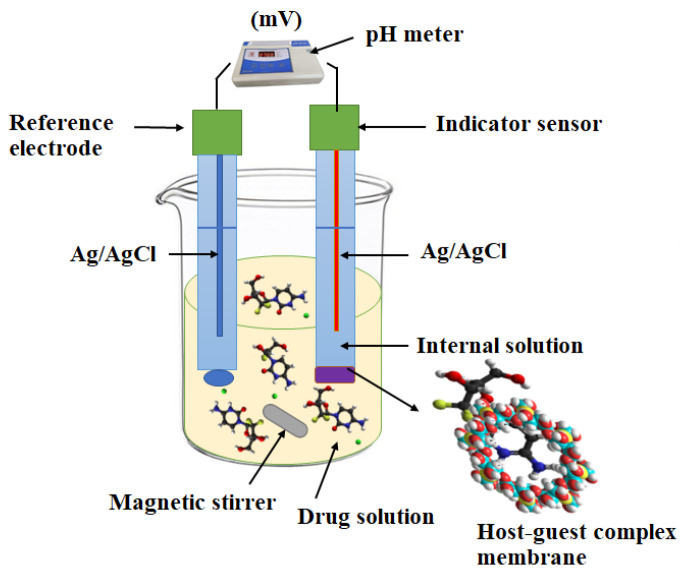
A general potentiometric system based on host–guest interaction.

By placing the GF sensors and a reference electrode within an electrochemical cell with an acetate buffer solution of pH 7, GF sensors were calibrated. After adding 1.0 mL of GF (1 × 10^−1^ to 1 × 0^−6^ mol L^−1^) to the measuring solution, the potential was recorded (Scheme 2). The calibration graphs were created by graphing the potential (E, mV) versus -log concentration following a stable measurement.

### 3.6. Determination of Gemifloxacin in Dosage Form

In a mortar, ten Fictive Tablets (each containing 320 mg of gemifloxacin mesylate) were crushed and combined. One tablet’s worth of crushed powder (320 mg of gemifloxacin mesylate) was precisely measured out, dissolved in water, and then filtered using Whatman filter paper after being sonicated for the appropriate period of time (10 min). The filtrate was then collected in a 100 mL volumetric flask and diluted to the proper strength with water. Appropriate aliquots (5.0 mL) were transferred to a volumetric flask of 50 mL, the pH was adjusted to 7 using an acetate buffer solution, and the mixture was completed with water. The examined GF sensors were used to record the pre-prepared sample potential.

## 4. Conclusions

The current work demonstrates the first host–guest recognition methodology-based electrochemical approaches for GF assessment in bulk and pharmaceutical dose form. Three PVC-based sensors that have been modified with β-CD and γ-CD and calixarene ionophores were developed for the specific detection of GF in which an inclusion complex formed. The sensors passed IUPAC verification and demonstrated good accuracy, precision, robustness, selectivity, and sensitivity. Calixarene (60 mV/decade) displays the best behavioral properties when compared to proposed sensors β-CD (55 mV/decade) and γ-CD (56 mV/decade). The developed methods have been effectively used to determine GF in bulk and dosage form. The statistical analysis utilizing the student *T*-test and F-test found no statistically significant difference between the reported and recommended techniques. In contrast, a potentiometric titration of GF with NaTPB was performed utilizing the indicated sensors as an indicator electrode.

## Figures and Tables

**Table 1:** Effect of ion-exchanger and ionophore on the electrode response.

Concentration, mg		Sensor 1	Sensor 2	Sensor 3
Lipophilic Ion	Carrier	Slope ± SD *		
2	25	47 ± 0.4	52 ± 0.5	52 ± 0.5
3	25	52 ± 0.5	53 ± 0.4	55 ± 0.4
5	25	55 ± 0.3	56 ± 0.4	60 ± 0.3
7	25	55 ± 0.3	56 ± 0.3	60 ± 0.3
9	0	43 ± 0.5	45 ± 0.5	60 ± 0.4
5	5	52 ± 0.5	52 ± 0.5	54 ± 0.5
5	10	52 ± 0.5	53 ± 0.4	55 ± 0.4
5	15	53 ± 0.4	54 ± 0.4	56 ± 0.4
5	20	55 ± 0.4	56 ± 0.3	60 ± 0.3
5	25	55 ± 0.4	56 ± 0.3	60 ± 0.3
5	30	55 ± 0.4	56 ±0.3	60 ± 0.3

* Slope ± SD (*n* = 3).

**Table 2:** The effect of plasticizer on the developed gemifloxacin sensors.

Plasticizer	DOP	DBS	o-NPOE
	Sensor 1		
Slope, mV	55	53	55
Response time, Sec	20	25	20
Calibration range, mol L^−1^	9 × 10^−6^–10^3^	9 × 10^−6^–10^−3^	8 × 10^−6^–10^−3^
	Sensor 2		
Slope, mV	55	53	56
Response time, Sec	20	20	20
Calibration range, mol L^−1^	8 × 10^−6^–10^−3^	9 × 10^−6^–10^−3^	8 × 10^−6^–10^−3^
	Sensor 3		
Slope, mV	58	55	60
Response time, Sec	20	25	20
Calibration range, mol L^−1^	8 × 10^−6^–10^−3^	9 × 10^−6^–10^−3^	8 × 10^−6^–10^−3^

**Table 3:** Selectivity coefficients the developed GF sensors.

Interfering Species	$K_{GF,\;B}^{Pot}\;\mathrm{Sensor}\;1$	$K_{GF,\;B}^{Pot}\;\mathrm{Sensor}\;2$	$K_{GF,\;B}^{Pot}\;\mathrm{Sensor}\;3$
Na^+^	1.1 × 10^−3^	0.9 × 10^−3^	1.2 × 10^−3^
K^+^	1.2 × 10^−2^	1.2 × 10^−3^	1.3 × 10^−3^
Ca^2+^	1.1 × 10^−2^	1.3 × 10^−3^	1.2 × 10^−3^
Fe^2+^	1.2 × 10^−2^	1.4 × 10^−3^	1.7 × 10^−3^
Magnesium Stearate	1.2 × 10^−3^	1.5 × 10^−3^	1.2 × 10^−3^
Acetate	1.3 × 10^−3^	1.3 × 10^−3^	1.3 × 10^−3^
Phosphate	1.3 × 10^−3^	1.3 × 10^−3^	1.3 × 10^−3^
Benzoate	1.2 × 10^−3^	1.3 × 10^−3^	1.3 × 10^−3^
Caffeine	1.2 × 10^−3^	1.3 × 10^−3^	1.3 × 10^−4^
Citrate	1.2 × 10^−3^	1.5 × 10^−3^	1.2 × 10^−3^
Glucose *	1.4 × 10^−3^	1.5 × 10^−3^	1.8 × 10^−3^
Lactose monohydrate *	1.4 × 10^−3^	1.5 × 10^−3^	1.8 × 10^−3^
Starch *	1.4 × 10^−3^	1.5 × 10^−3^	1.7 × 10^−3^
Microcrystalline cellulose *	1.4 × 10^−3^	1.4 × 10^−3^	1.6 × 10^−3^

* match potential method.

**Table 4:** Analytical characteristics of GF-sensors.

Parameter	Sensor 1	Sensor 2	Sensor 3
Slope, (mV decade^−1^)	55	56	60
Intercept, mV	239 ± 0.5	244 ± 0.5	240 ± 0.5
Correlation Coefficient, (r^2^)	0.999	0.998	0.999
Calibration rang, M	8 × 10^−6^–1 × 10^−2^	9 × 10^−6^–1 × 10^−2^	8 × 10^−6^–1 × 10^−2^
LOQ, mol L^−1^	8 ×10^−6^	9 × 10^−6^	8 ×10^−6^
LOD, mol L^−1^	2.40 × 10^−6^	2.70 × 10^−6^	2.42 × 10^−6^
Response time, sec	20 ± 0.5	20 ± 0.5	20 ± 0.5
pH range	6–9	6–9	6–9

**Table 5:** Day and inter-day recovery and precision of gemifloxacin using the proposed sensors.

Concentration (µg mL^−1^)	During a Day		
	Sensor 1	Sensor 2	Sensor 3
	Recovery, % ± RSD% *	Recovery, %± RSD%	Recovery, % ± RSD%
5.0	97.5 ± 2.7	97.5± 2.8	97.5 ± 2.8
25	98.0 ± 2.7	97.5 ± 2.6	97.5 ± 2.7
100	98.5 ± 2.0	98.0 ± 1.9	98.0 ± 1.9
200	99.0 ± 1.8	98.0± 1.8	98.0 ± 1.8
300	99.0 ± 1.8	98.0 ± 1.7	99.0 ± 1.8
3000	99.0 ± 1.7	99.0 ± 1.7	99.0 ± 1.8
Within-days			
5	97.5 ± 2.9	97.5 ± 2.8	97.5 ± 2.9
25	97.6 ± 2.8	97.5 ± 2.7	97.5 ± 2.6
100	98.0 ± 2.6	97.5 ± 2.5	97.5 ± 2.5
200	98.5 ± 2.3	98.0 ± 2.5	98.0 ± 2.2
300	98.5 ± 2.3	98.0 ± 2.3	98.0 ± 2.2
3000	99.0 ± 2.3	98.0 ± 2.3	99.5 ± 2.3

Average of 5 measurements ± RSD. * RSD%, Relative standard deviation %.

**Table 6:** Comparison of the investigated sensors with reported potentiometric sensors.

Ion-Pair	Calibration Range	Matrix	Slope, mV	Measuring Medium	Ref.
GF-reineckate	1 × 10^−5^–1 × 10^−2^	PVC	20	Aqueous	26
Hydroxypropyl-β-cyclodextrine	0.5–1000 μM	ZnO nanoroad	33	Phosphate buffer	27
GF-PM GF-PT	1 × 10^−7^–1 × 10^−2^	Coated-wire	55	Aqueous	28
β-CD	8 × 10^−6^–1 × 10^−2^	PVC	55	pH 7	This work
γ-CD			56		
Calixarene			60		

**Table 7:** Direct determinations of GF in bulk form using the proposed sensors.

Concentration Added (µg mL^−1^)	Recovery ± RSD Sensor 1	Recovery ± RSD Sensor 2	Recovery ± RSD Sensor 3
5	97.5 ± 2.8	97.0 ± 2.8	97.5 ± 2.7
25	97.5 ± 2.6	97.5 ± 2.5	97.5 ± 2.5
50	98.0 ± 2.4	98.0 ± 2.4	98.0 ± 2.3
100	98.5 ± 2.1	98.5 ±2.0	98.5 ± 2.0
200	99.5 ± 2.0	98.5 ±2.0	98.5 ±2.0
300	99.0 ± 1.8	99.0 ± 2.0	99.0 ± 1.8
3000	99.0 ± 1.8	99.0 ± 1.8	99.0 ± 1.7

*n* = 5 ± RSD.

**Table 8:** Determination of gemifloxacine in its dosage from.

Preparation	Sensor 1 Recovery ± RSD	Sensor 2 Recovery ± RSD	Sensor 3 Recovery ± RSD	Reported Method [40]
FACTIVE 320 mg	98.5 ± 2.9	98.0 ± 2.85	99 ± 2.80	98.5 ± 2.8
*t* test	0.10	0.098	0.163	
F test	1.10	1.12	1.56	

The mean of five determinations.

## Data Availability

All data in the manuscript are available from all authors.

## Sample Availability

Samples of the compounds are available from the authors.
